# Current rates and mechanisms of subsea permafrost degradation in the East Siberian Arctic Shelf

**DOI:** 10.1038/ncomms15872

**Published:** 2017-06-22

**Authors:** Natalia Shakhova, Igor Semiletov, Orjan Gustafsson, Valentin Sergienko, Leopold Lobkovsky, Oleg Dudarev, Vladimir Tumskoy, Michael Grigoriev, Alexey Mazurov, Anatoly Salyuk, Roman Ananiev, Andrey Koshurnikov, Denis Kosmach, Alexander Charkin, Nicolay Dmitrevsky, Victor Karnaukh, Alexey Gunar, Alexander Meluzov, Denis Chernykh

**Affiliations:** 1 National Tomsk Research Polytechnic University, 30 Prospect Lenina, Tomsk, Alaska 634050, Russia; 2 International Arctic Research Center, University of Alaska Fairbanks, Akasofu Building, Fairbanks, Alaska 99775-7320, USA; 3 Pacific Oceanological Institute, Russian Academy of Sciences, 43 Baltiiskaya Street, Vladivostok 690041, Russia; 4 Department of Environmental Science and Analytical Chemistry, and the Bolin Centre for Climate Research, Stockholm University, Stockholm 10691, Sweden; 5 Institute of Chemistry, Russian Academy of Sciences, 100-Letiya Vladivostoka, Vladivostok 690022, Russia; 6 P.P. Shirshov Oceanological Institute, Russian Academy of Sciences, 36 Nahimovski Prospect, Moscow 117997, Russia; 7 Moscow State University, 1-12 Leninskie Gory, Moscow 119991, Russia; 8 Institute of Geography, Russian Academy of Sciences, 29 Staromonetniy Pereulok, Moscow 119017, Russia; 9 University of Tyumen, 6 Volodarskogo Street, Tyumen 625003, Russia; 10 Melnikov Permafrost Institute, Russian Academy of Sciences, 36 Merzlotnaya Street, Yakutsk 677010, Russia

## Abstract

The rates of subsea permafrost degradation and occurrence of gas-migration pathways are key factors controlling the East Siberian Arctic Shelf (ESAS) methane (CH_4_) emissions, yet these factors still require assessment. It is thought that after inundation, permafrost-degradation rates would decrease over time and submerged thaw-lake taliks would freeze; therefore, no CH_4_ release would occur for millennia. Here we present results of the first comprehensive scientific re-drilling to show that subsea permafrost in the near-shore zone of the ESAS has a downward movement of the ice-bonded permafrost table of ∼14 cm year^−1^ over the past 31–32 years. Our data reveal polygonal thermokarst patterns on the seafloor and gas-migration associated with submerged taliks, ice scouring and pockmarks. Knowing the rate and mechanisms of subsea permafrost degradation is a prerequisite to meaningful predictions of near-future CH_4_ release in the Arctic.

## Abstract (web summary)

The rate of subsea permafrost degradation is a key factor controlling marine methane emissions in the Arctic. Here, using re-drilled boreholes, the authors show that the ice-bonded permafrost table in the near-shore East Siberian Arctic Shelf has deepened by ∼14 cm per year over the past 31–32 years.

Arctic coastal zone permafrost (ground that remains ≤0 °C for ≥2 year) developed when the Northern Hemisphere cooled ∼2.5 Myr ago^1^. Most subsea permafrost formed on the continental shelves when the shelves were exposed during periods of low sea level associated with times of major glacial activity^2^. As the glaciers eventually melted, the sea level rose, which submerged this permafrost^3^. Inundation can markedly change permafrost properties because the permafrost is warmed by as much as 17 °C by the overlying seawater^4^. The following factors were suggested to determine the evolution of subsea permafrost after inundation: duration of submergence compared with the duration of previous exposure above the sea surface; thermal state and thickness of permafrost before inundation; coastal morphology and hydro- and lithodynamics; shoreline configuration and retreat rate; pre-existing thermokarst (that is, the process by which characteristic landforms result from the thawing of ice-rich permafrost or the melting of massive ice) accompanied by formation of thaw lakes; bottom water temperature and salinity; and sediment composition, including ice content^5^,^6^,^7^,^8^.

Warming of the East Siberian Arctic Shelf (ESAS) began ∼12–13 thousand years (kyr) ago when the entire shelf area was exposed above sea level, forming a major fraction of the coastal plain^7^. By the time of inundation, numerous thaw lakes underlain by taliks had developed in that area due to thermokarst^9^. A talik is a layer or body of unfrozen ground in a permafrost area in which the temperature is above 0 °C due to the local thermal regime of the ground^10^. The fate of these thermokarst-induced features in the near-shore zone, only recently inundated, has long been debated^8^,^9^,^11^,^12^. The widely accepted hypothesis is that the <0 °C bottom seawater temperature would halt thermokarst formation and cause taliks to freeze by creating a negative temperature profile in the sediments^7^,^8^,^9^. However, no observational evidence to confirm this hypothesis has existed to date.

On the contrary, some authors suggested that seawater could be transported into the sediments at sufficient rates to lower the freezing point of the sediment pore water, even in ice-bonded permafrost^13^,^14^. In addition, via convective fingering, seawater transport rates could be orders of magnitude greater than heat conduction from the surface^15^, perhaps preventing freezing of the submerged thaw-lake taliks and, thus, causing advanced top–down permafrost degradation^16^. In addition, the ESAS near-shore zone is largely affected by riverine runoff, which causes the mean annual bottom seawater temperature to be >0 °C (ref. 17). Heat flux from large rivers can cause deep talik formation beneath riverbeds; it has been suggested that such taliks might exist below the paleo rivers^18^. A significant area of the ESAS is affected by paleo-river valleys^19^. A substantial part of submerged ESAS permafrost consists of ice complexes (ICs), which are Late Pleistocene ice-rich syncryogenic deposits with massive ice wedges^20^,^21^. Before inundation, ICs are subjected to two destructive processes: thermo-denudation (upslope permafrost retreat under the influence of insolation and heat flux on the slope) and thermo-abrasion (mechanically and thermally caused retreat of exposed permafrost due to seawater and wind erosion)^22^,^23^. Some authors believe that after ICs are submerged, they are subjected to thermo-abrasion and chemical- and current-induced seafloor erosion, to list a few such destructive processes^24^,^25^.

Permafrost-degradation rates could be evaluated by assessing changes in the ice-bonded permafrost table (IBPT) position. The position of the IBPT in the ESAS has been investigated using seismic techniques^26^,^27^. However, there are problems with high attenuation of the reflected seismic signal where sediments contain gas^28^ and/or reflect variability in permafrost properties^29^,^30^. Methods based on electrical properties of frozen/unfrozen ground were shown to be applicable in shallow coastal waters^31^,^32^. Poor knowledge of the physical and chemical processes occurring within subsea permafrost, combined with a lack of observational data for model calibration, restricts further progress in modelling the current state of subsea permafrost and associated methane (CH_4_) releases in the ESAS^16^,^33^. It is, therefore, necessary to conduct comprehensive geocryological investigations.

This study aimed to document subsea permafrost-degradation rates after submergence by directly studying frozen ground samples recovered from drilled boreholes, and interpreting geophysical data collected during repeated observations in the study area. On the basis of results of first comprehensive scientific re-drilling investigation of subsea permafrost in the ESAS, here we present observation-based demonstration of thawing of subsea permafrost resolved over decadal scale. Interpretation of geophysical data calibrated by drilling allows resolving on inter-annual scale upward migration of shallow gas. We demonstrate that thermokarst occurs after inundation, submerged thaw lakes not always freeze and could serve as gas-migration paths, and ice scouring serves as important mechanism of permafrost disturbance associated with gas releases. Knowing the rate and mechanisms of subsea permafrost degradation is a prerequisite to meaningful predictions of near-future CH_4_ release on the Arctic shelf.

## Results

### Study area

**Figure 1:**
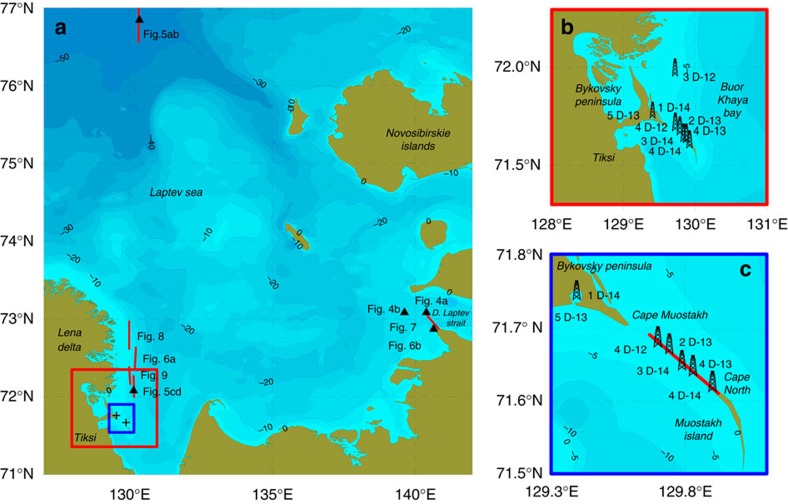
Study area bathymetry and position of the investigated sites. (**a**) Red and blue rectangles mark study areas, where drilling was conducted in 2011–2014, position of the sites investigated in marine expeditions, data from which are presented in Figs 4, 5, 6, 7, 8, 9 are shown as black triangles (2D sites) and red lines (transects); two black crosses in the blue rectangle show position of the drilling transect conducted in 2012–2014 (shown enlarged in **b**,**c**); (**b**) position of the boreholes drilled in March 2011–2013; (**c**) enlarged position of the drilling transect performed the northern part of MI in 2012–2014.

**Figure 2:**
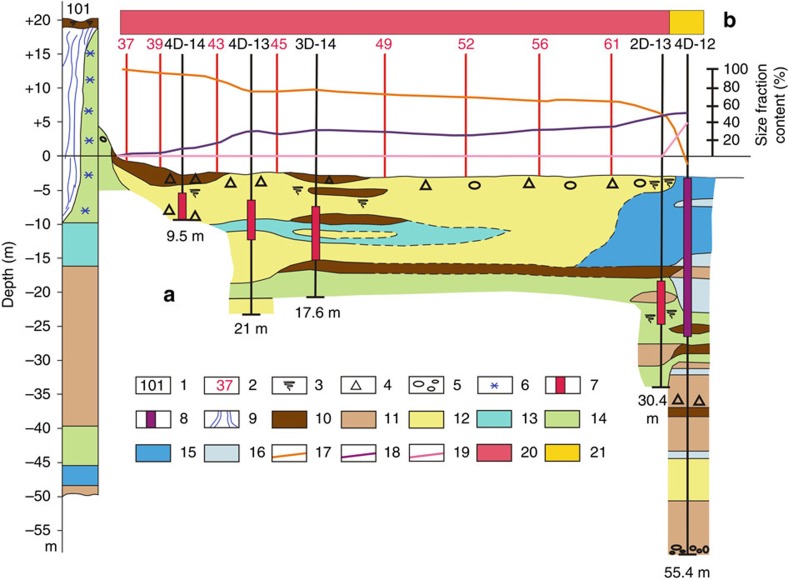
Geomorphological structure of the sediments along the drilled transect in the near-shore zone of the ESAS. (**a**) Cross section from Cape North, MI towards Cape Muostakh, Bykovsky Peninsula with marked positions of the re-drilled boreholes. Boreholes 4D-14 (former 301), 4D-13 (former 303), 3D-14 (former 304) and 2D-13 (former 305) are re-drilled boreholes; borehole 4D-12 is the borehole drilled for the first time. Red spikes identify changes in the position of the IBPT in drilled boreholes during the last 31–32 years; purple spike in borehole 4D-12 show change in the position of the IBPT since the time of inundation. The top of the red/purple spike marks the former top of the IBPT; the low end of the spike marks the recently observed IBPT; numbers below each borehole show the length of recovered sediment cores. (**a**) 1—marks of drilled boreholes; 2—marks of sediment sampling sites; 3—plant remains within sediments; 4—gravel; 5—pebbles; 6—frozen ground in the coastal IC; 7—change in position of the IBPT during the last 31–32 years (boreholes 4D-14, 4D-13, 3D-14 and 2D-13); 8—change in position of the IBPT in borehole 4D-12 (since inundation); 9—wedge-like structure of ice in the coastal IC; 10—medium sand; 11—fine sand; 12—silty sand; 13—sandy silt; 14—clayey silt; 15—silty clay; 16—clay; (**b**) shelf-ward dynamics in grain-size distribution in sediments collected at 5 cm depth at selected sites along the transect (weight-% of major size fractions): 17—change in contribution of sands (>0.1 mm); 18—change in contribution of silt (0.1–0.01 mm); 19—change in contribution of clay (<0.01 mm); shift in sedimentology from predominant contribution of large-medium grain-size fractions (20) to medium-fine grain-size fractions (21).

In 2008–2014, we conducted four marine expeditions and four drilling campaigns in the study area between 70–74° N and 129–131° E with focus on the near-shore Laptev Sea southeast of the Lena Delta, the Buor-Khaya Bay (BKB, between 70–74° N and 129–131° E), and the Dmitry Laptev Strait (DLS, between 72.5–73.5° N and 138–143° E, Fig. 1). In drilling campaigns, we investigated the thermal regime, geomorphology, lithology and geocryology of sediment cores extracted from drilled boreholes and sediments sampled along the drilling transect (Fig. 2). We also performed few geoelectrical surveys, results of which were validated by direct measurement of electrical resistivity of recovered sediments. In marine expeditions, we collected conductivity-temperature-depth (CTD) data, performed high-resolution sub-bottom profiling, sonar-derived imagery and visual observations (using an autonomous underwater vehicle) of geomorphological features of the seafloor (subsea thermokarst, ice scouring and pockmarks) associated with gas releases.

To assess modern rates of downward permafrost degradation, we re-drilled four boreholes first drilled in 1982–83 (ref. 20) and also drilled one new borehole northwest of Muostakh Island (MI); MI represents remains of the Bykovsky Peninsula submerged <0.5 kyr ago^11^. Water depths vary from 2.5 to 3.4 m. Bottom sediments are predominantly silty sands. The seafloor here represents an abrasion-accumulative terrace of the former coastal plain formed via inundation about 2.7–3 kyr ago^21^. MI IC thickness is 31 m, 10 m of which extends below sea level^21^. At the time of deposition, the IC temperature varied from −25 to −28 °C; the current MI permafrost temperature is −10.4 °C (ref. 23). Sand and silt are the predominant sediment types observed in the area; ice-poor sands of Pliocene-early Pleistocene age underlay the IC deposits^20^. Mechanical denudation processes dominate lithogenesis in this area; ice transport, bottom erosion, and sedimentary matter resuspension and redistribution are widespread^34^. This area has been strongly affected by global warming^17^,^23^, and exhibits a high coastal erosion rate, which has approximately doubled during the last 62 years^23^,^35^. During the last 5 kyr, after the glacio-eustatic sea level reached its highstand at the ∼30 m isobaths in the studied area, the shallower near-shore zone submergence has been occurring primarily via erosional processes^36^,^37^.

### Lithology and thermal regime of sediment cores

Five boreholes (4D-14, 4D-13, 3D-14, 2D-13 and 4D-12) were drilled along the transect located 300–2,900 m from Cape North, MI and extending towards Cape Muostakh of the Bykovsky Peninsula (Fig. 2a). Borehole 4D-14 was drilled 300 m from Cape North, MI; a 9.5 m sediment core was recovered. An IBPT was identified at 8.6 m below sea level (b.s.l.). The lithological structure of the thawed sediments in borehole 4D-14 was uniform (Supplementary Fig. 1) and consisted of light grey/grey coarse sand with grain-size fraction >0.1 mm composing 99% of sediments (Fig. 2b). The cryostructure of the frozen part of the 4D-14 sediment core was massive, which could represent the ice wedge of the submerged coastal IC (Supplementary Figs 2 and 3).

**Figure 3:**
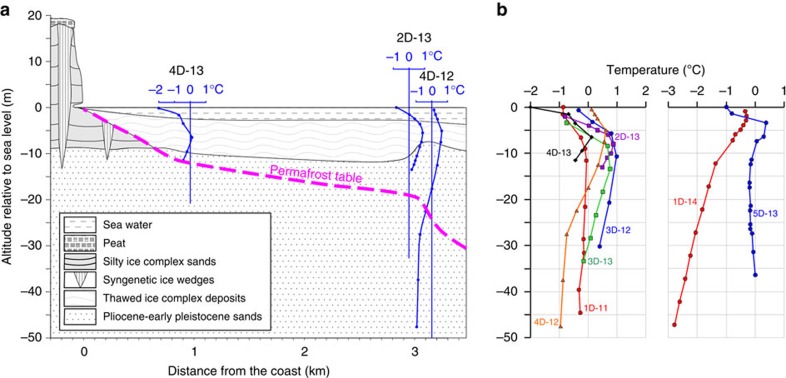
Thermal regime of subsea permafrost based on results of direct observations. (**a**) Data show that along the re-drilled transect, sediment temperatures in boreholes 4D-13, 2D-13 and 4D-12 varied from −2 to +1 °C. (**b**) Examples of thermal state of sediment cores recovered from a few boreholes in 2011–2014; the results show that sediment temperatures varied from −3  to +1 °C, increasing downwards in some cores.

Borehole 4D-13 was drilled 600 m from Cape North, MI. The sediment core recovered from the borehole was 21 m long and was composed of five units (Supplementary Fig. 1). The uppermost 6 m is characterized by partly bedded brown coarse-grained silty sand, which is underlain by 1.8 m of sandy silt followed by 10.6 m of partly bedded medium-grained silty sand, which included plant and wood remains. Below, there was a 1.1 m layer of clayey silt with organic inclusions. The lowermost 1.5 m layer was composed of partly bedded silty sand containing plant and wood remains. The IBPT in borehole 4D-13 was identified at 11.4 m b.s.l.; the temperature of the unfrozen part of the sediment varied from −1 to 0.1 °C (Fig. 3a), and was interpreted as thawed/cryotic (thawed is unfrozen at temperatures >0 °C; cryotic is unfrozen at temperatures <0 °C). Cryology of the 4D-13 sediment core below the IBPT was characterized as mostly non-structured ice within frozen sediments, which in places included fine lenticular ice, predominantly within silty sand (Supplementary Figs 2 and 3).

Borehole 3D-14 was drilled 850 m from Cape North, MI; the recovered sediment core was 17.6 m long. The lithological structure of the sediment core was heterogeneous (Supplementary Fig. 1). The uppermost 1 m of sediments consisted of coarse/medium grey sand; this layer was underlain with 3.8 m of bedded silty sand interlayered with two thin layers (∼0.2 m) of medium-grained sand. Below, there was a 1 m layer of sandy silt followed by 2.4 m of medium-grained sand; the next unit (3.4 m) consisted of partially bedded sandy silt with organic inclusions and inclusions of plant remains. Below, there was a 3.6 m layer of silty sand followed by a 2 m layer of clayey silt containing organic inclusions. The IBPT was identified at 12.8 m b.s.l. The cryostructure of the sediment core below the IBPT was identified as mostly consisting of inclined lenticular ice; 0.5–2 mm ice lenses were included in the sediments (Supplementary Figs 2 and 3).

Borehole 2D-13 was drilled 2,500 m from Cape North, MI; a 30.4 m-long sediment core was recovered from the borehole (Supplementary Fig. 1). The uppermost 4.5 m of the sediment core consisted of medium- to fine-grained silty sand with lenses of coarse-grained sand and gravel included in the sediments. Below was 10.3 m of dark grey, greenish or black silty clay, which included lenses of plant remains and organic inclusions. The next unit was composed of 2.5 m of medium- to fine-grained sand, dark grey in colour; this unit was followed by 11.5 m of clayey silt, dark grey or greenish, which included organic inclusions, plant detritus and wood remains. The lowermost layer consisted of 1.6 m of silty sand, dark grey in colour. The IBPT was identified at 19.3 m b.s.l.; the cryostructure below the IBPT was ice-rich and predominantly non-structured with rare inclusions of lenticular ice and small lenses <0.1 mm in size; between these two layers we identified a layer (0.5 m) of cryotic sediments. The temperature of unfrozen sediments varied from 0 to 1 °C; these sediments were identified as thawed sediments interlayered with cryotic sediments (Fig. 3a; Supplementary Figs 2 and 3).

Borehole 4D-12 was drilled 2,900 m from Cape North, MI; the sediment core recovered from the borehole was 28 m long (Supplementary Fig. 1). The lithology of the upper 20 m of this core consists of multiple layers of silty clay and clay interspersed with thin layers (<1 m) of clayey sand (at 9.5 m b.s.l.) and clayey silt (at 10.6 m b.s.l.). Below, we identified a 4.5 m layer of medium-grained sand followed by 0.6–0.9 m layers of silty clay, clay, sandy clay and clayey sand. All layers included plant and wood remains and gravel. The IBPT was identified at 26.4 m b.s.l.; the cryostructure of the sediment core below the IBPT was massive and, at some depths, was interlayered by non-structured ice. The temperature of unfrozen sediments varied from −0.5 to 0.7 °C, and sediments were interpreted as remaining in a cryotic/thawed state (Supplementary Figs 2 and 3). The thermal regime in the three re-drilled boreholes presented in Fig. 3a is compared with the thermal state of the few other boreholes drilled in the near-shore ESAS area in 2011–2014 (Fig. 3b; other results from these boreholes are not presented in this paper). Volumetric ice content of different cryostructures identified in the drilled boreholes is presented in Supplementary Table 1.

### Rates of permafrost degradation

To elucidate modern rates of downward permafrost degradation, we compared the position of the IBPT in four re-drilled cores with the position of the IBPT in four boreholes first drilled in 1982–83 at the same locations. The boreholes were localized using a shore-based theodolite technique (see Methods). The IBPT positions observed in 1982–1983 at sites 301, 303, 304 and 305 were at 3.3–4.2 m, 5.8–7 m, 8.3–8.6 m and 16–16.8 m b.s.l., correspondingly (Table 1). In 2012–2013, the IBPT positions were identified at 8.6 m, 11.4 m, 12.8 m, and 19.3 m b.s.l. at sites 4D-14 (former 301), 4D-13 (former 303), 3D-14 (former 304), and 2D-13 (former 305), correspondingly. IBPT deepening during the last 31–32 years varied from 9.3 to 18.3 cm year^−1^ with a mean rate of 14±3.1 cm (mean±s.e.m.) per year during the last 31–32 years. Position of the IBPT between drilled boreholes was established based on results of geoelectric investigation performed along the drilling transect. Sediment temperature, lithology and geochemistry were used to convert recorded electrical resistivity to possible ice content of sediments along the drilled transect (Supplementary Fig. 4; Supplementary Table 2).

To assess rates of permafrost degradation before 1982, we calculated the time since inundation by applying an empirical modelling approach based on modern bathymetry and observational time series of coastal erosion. Following Bauch *et al*.^36^, we assume that by 5 kyr ago, sea level in this region achieved its highstand at the 30 m isobath. After that, coastal zone inundation occurred due to the erosion-based processes of thermo-denudation and thermo-abrasion^37^, implying that between two subsequent isobaths, the speed at which water depth adjusted to existing sea level was proportional to coastal erosion rates (Supplementary Figs 5 and 6). The mean coastal erosion rate was set based on observational data collected at regular MI stations, where coastal erosion has been monitored over the last few decades^11^,^35^. These time series documented that from 1983 to 2013 the coastline position shifted inland by ∼180 m, establishing a mean annual coastal erosion rate of 6 m year^−1^. We calculated that IBPT deepening rates from the time of inundation until 1982–1983 varied from 3.9 to 8.5 cm year^−1^ with a mean rate of 5.7±2.8 cm year^−1^, less than half as much as IBPT deepening rates observed during the last 31–32 years (Table 1). Calculations of pre-1982 rates of permafrost degradation are more uncertain than those observed during the last three decades, and reflect the current state of knowledge on this topic (Supplementary Discussion).

We found in our study that the further we travelled from the coast, the deeper we identified the position of the IBPT; at the same time, rates of permafrost degradation during the last 30 years were higher in boreholes 4D-14, 4D-13 and 3D-14 located closer to the coast. Indeed, the position of the IBPT changed from 8.6 m b.s.l. in borehole 4D-14, which was 300 m from Cape North, MI to 19.3 m b.s.l. in borehole 2D-13, which is 2,500 m from the coast; rates of permafrost deepening decreased from 18.3 cm year^−1^ in borehole 4D-13 to 9.3 cm year^−1^ in borehole 2D-13. The position of the IBPT in the drilled boreholes often coincided with the boundary between coarse- to medium-grained sediments (sands-silts) and medium- to fine-grained sediments (silt-clay). A specific feature of the lithology of the boreholes drilled closer to the coast (4D-14, 4D-13 and 3D-14) is that the uppermost sediment layers (5 cm from the top) in these boreholes were composed of sands, which represent remains of ICs of Pleistocene age; thus, in these boreholes either no Holocene-age accumulations occurred or erosion-driven processes balanced them. In the uppermost layers, sands with grain size >0.1 mm composed 70 to 99% of the total sediment weight, while fractions of silt (0.1–0.01 mm) and clay (<0.01 mm) was <30%. This distribution of grain size was different from that in boreholes further from the coastline (2D-13 and 4D-12) in which the fraction of finer sediments with grain size <0.1 mm increased to 50–95% (Fig. 2b).

### Specific features of seafloor morphology

**Figure 4:**
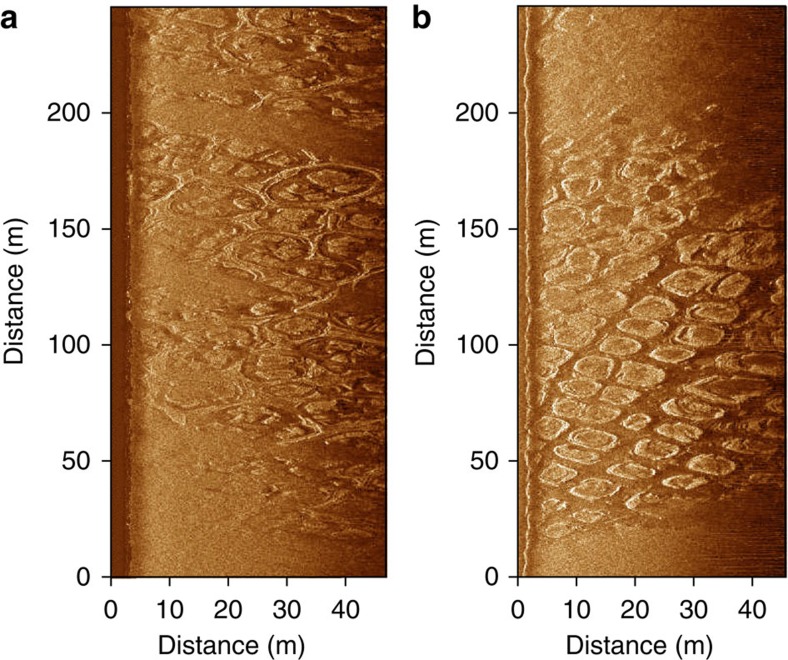
Two types of seafloor polygonal structure observed in the study area. (**a**) A high-resolution side-scan sonar image showing a type of polygonal structure, which represents a regularly shaped depression surrounded by a raised shaft; (**b**) a high-resolution side-scan sonar image showing a type of polygonal structure characterized by a regular pattern consisting of raised oval-like shapes.

We used high-resolution reflection seismic and sonar-derived imagery to identify specific seafloor morphology features that could reflect ongoing permafrost-degradation processes such as thermokarst. When on-land permafrost thaws, it leaves behind a regularly shaped hummocky landscape that resembles a polygonal pattern^4^,^7^. In the study area, we frequently observed such a pattern on the seafloor (Fig. 4). Because the pattern was not buried by Holocene sediments, we suggest that the seafloor appearance reflects modern thermokarst processes, which keep developing after permafrost submergence. However, a polygonal pattern characteristic of thermokarst, as observed in the near-shore area of the ESAS, could also represent a relic feature and remain unburied by sediments due to the predominantly erosional character of sedimentological processes in some near-shore areas of the ESAS.

**Figure 5:**
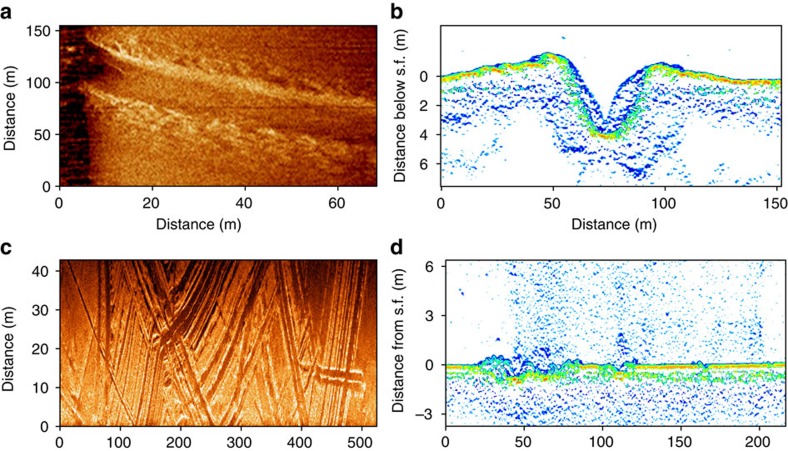
Ice scouring and associated features observed in the ESAS. (**a**) A high-resolution side-scan sonar image of an ice scour observed in the seafloor; (**b**) a vertical profile of the ice scour as it occurs on the high-resolution sub-bottom profile image in one location. The depth of the depression created by ice scour reaches ∼4 m; (**c**) a high-resolution side-scan sonar image demonstrating wide occurrence of ice scours in the seafloor in the far offshore area of the ESAS; (**d**) a high-resolution sub-bottom profile image showing multiple vertical profiles of the ice scours and bubble plumes propagating from the seafloor in the area of investigation.

**Figure 6:**
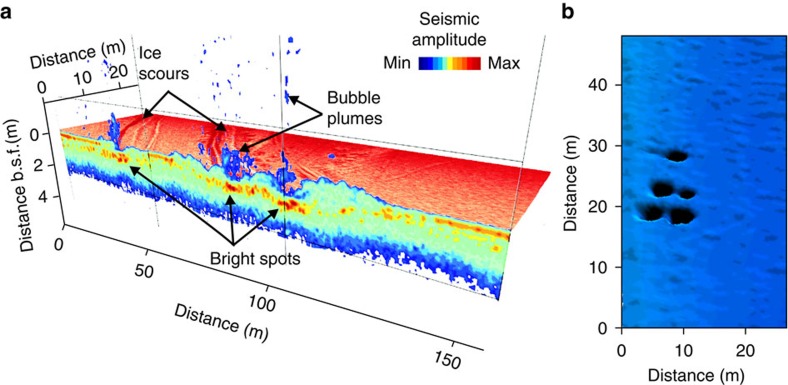
Specific morphological features of the seabed and the seafloor observed in the study area. (**a**) Three-dimensional coloured view of sub-bottom morphology based on interpretation of high-resolution sub-bottom profile data (vertical profile) combined with side-scan backscatter data; colour variations refer to the amplitude response of the seabed (maximum shown in red, minimum shown in blue). Morphological features within sediments with highest amplitudes are shown as bright spots (red-orange colours). As seen from the image, bubble releases to the water occur where ice sours unroof shallow gas accumulations in the seabed; b.s.f. refers to below seafloor; (**b**) a view of multi-beam sonar backscatter data used to detect typical pockmarks in the seafloor in the study area where submerged thaw lake was identified as shown in Fig. 7.

Another permafrost destabilization mechanism, specific to the shelf system, is ice scouring, which mechanically disintegrates the upper frozen and/or thawed sediment layers^38^,^39^. Ice scouring was observed as a ubiquitous morphological feature, not only of the shallow part of the ESAS but also offshore (Figs 1a and 5a–c). Ice scouring is evidenced by a long, linear, relatively-straight furrow a few tens of metres wide extending for many tens of kilometres^40^. In the ESAS, ice scouring penetrated as much as 10 m into the sediments, and where surface sediments were underlain with free gas, strong ebullition to the water column through the scours was observed (Fig. 5d). Ice scouring likely provides an important mechanism, not only for unroofing shallow gas accumulations (Fig. 6a), but also for allowing gases to avoid anaerobic oxidation due to removal/disintegration of the uppermost sediment layers, where anaerobic oxidation of CH_4_ occurs within the sulphate-reduction zone.

### The fate of submerged taliks

The fate of gas-migration pathways such as thaw-lake taliks that formed before ESAS inundation was assessed by repeated geophysical surveys in the Dmitry Laptev Strait (DLS) specifically in regions of high dissolved CH_4_ concentrations, called ‘hot spots’^41^ (here between 72.5–73.5° N and 138–142° E, Fig. 1a). Such hot spots suggest elevated sediment permeability for ascending gas, which could only be possible if deep taliks serve as gas-migration pathways within permafrost. Despite the fact that this area was submerged relatively recently, <<1 kyr ago, which is insufficient time for upwardly developing taliks to penetrate through the entire permafrost body and reach the permafrost top^7^, significant variations in position of the IBPT (>120 m) in the recovered sediment cores were observed during the drilling campaign performed in the DLS in the 1980s (ref. 5).

**Figure 7:**
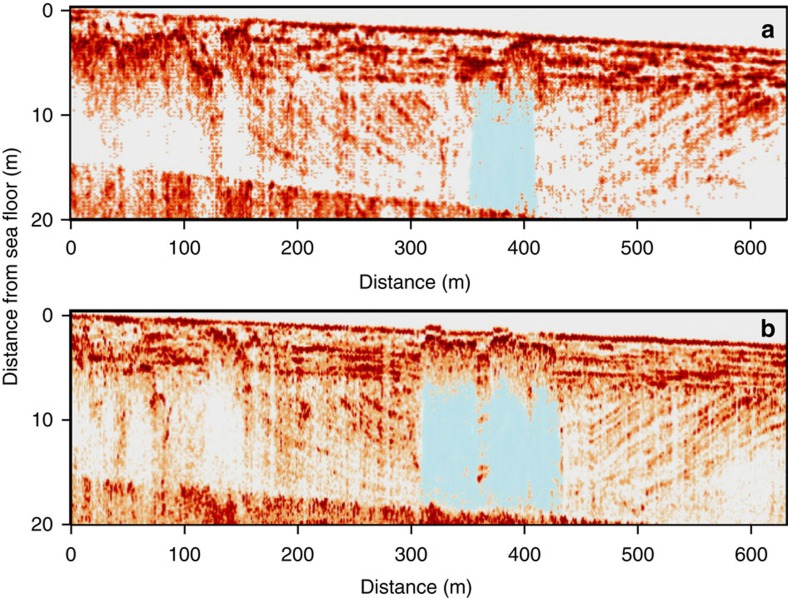
High-resolution sub-bottom profile images of gas plumes propagating through the centre of a submerged lake basin in 2008 and 2011. (**a**) Column-like acoustic anomalies (blanked areas) interpreted as gas plumes are shown as areas with no colour and blue colour. Acoustic anomalies highlighted in blue correspond with the centre position of the submerged lake basin and interpreted as gas propagating through the submerged thaw-lake talik. In 2008, the top boundary of this acoustic anomaly was located ∼5 m below the seafloor; (**b**) in 2011, the position of the top boundary of this acoustic anomaly was reaching the seafloor, causing doming of the surface layer of sediments. During the survey in 2011, bubble plumes of CH_4_ releasing to the water column from the seafloor were observed at this site; multiple pockmarks in the seafloor were also documented within the site as shown in Fig. 6b.

In our investigation of DLS, interpretation of high-resolution seismic imagery allowed identification of a presumed submerged thaw-lake basin beneath the seafloor with a central washed-out zone (Fig. 7). Repeated observations along the same transect 3 years later revealed a gas front moving upwards, because the top gas front boundary changed from ∼3 m below the seafloor in 2008 to the surface of the seafloor in 2011. Hydro-acoustical images of gas bubbles released from doming surface sediments were recorded, and increased concentrations of dissolved CH_4_ in the water column (≤120 nM) were measured. We also observed visible morphological signs of recent gas releases (pockmarks) within the site (Fig. 6b). On the basis of our data, we suggest that submerged thaw-lake taliks may not freeze; instead, they may keep developing, creating pathways for ascending gas. Such pathways propagating throughout the entire permafrost body could allow vigorous CH_4_ venting, if over-pressured gas reservoirs beneath are unroofed.

Another possible mechanism for preventing taliks from freezing and/or causing talik formation could be groundwater flow through coastal sediments, especially in the areas underlain with the faults. This could cause formation of so-called tectono-genic taliks^42^. These taliks could be identified by groundwater discharge that could be manifested as large point sources, which are temporally and spatially variable and could have a significant impact on the geochemical parameters of coastal waters^43^. Groundwater is usually terrestrially derived and enriched in naturally occurring radionuclides such as, ex^224^Ra and ^223^Ra, relative to seawater^44^. Releases of groundwater, including intra-permafrost water, might lead to formation of taliks within subsea permafrost, as we observed in one near-shore area, where a geoelectric survey showed spots of low electrical resistivity within the study area characterized by high resistivity of the sediment (Supplementary Fig. 7). High concentrations of ex^224^Ra and ^223^Ra (19 and 0.65 dpm/100 l^−1^ versus <0.1 and <0.05 dpm/100 l^−1^ in normal seawater) and thermohaline anomalies (higher temperature and lower salinity of seawater) were detected in the bottom water, which suggests groundwater discharge at this location. The location of this site coincides with the position of the fault^45^, which could have resulted in the development of a tectono-genetic talik.

### Gas movement through sediments

**Figure 8:**
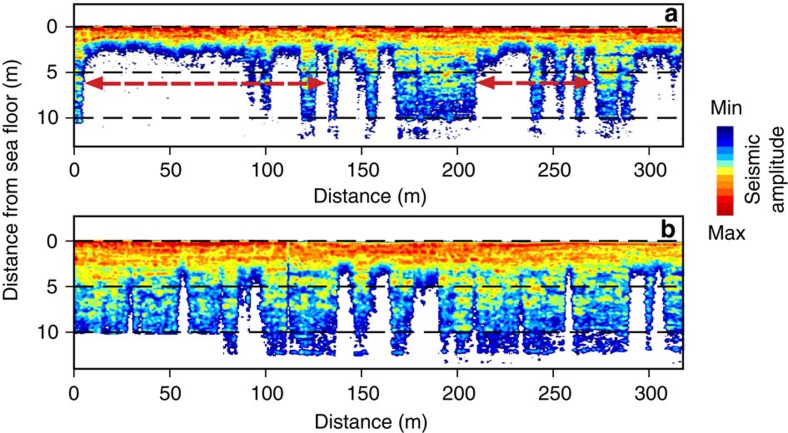
Upward movement of gas front observed in the same area of investigation in two subsequent years. (**a**) A high-resolution sub-bottom profile image is interpreted based on colour variations referring to change from maximum seismic amplitudes (shown in red) to minimum amplitudes (shown in blue). Blanked (or washed-out, shown in white) zones are interpreted as attenuated and scattered by gas bubbles (acoustic turbidity)^28^,^46^. The upper boundary of the blanked zone indicate position of the gas front in 2012, ∼2 m below the seafloor; red arrows indicate areas of most pronounced upward movement, ≤5 m during 1 year; (**b**) a high-resolution sub-bottom profile image shows that the upper boundary of the blanked zones in 2011 is located ≤10+ m below the seafloor, in the same areas marked with red arrows in **a**; blanked or semi-blanked acoustic anomalies are interpreted as gas-charging sediments based on validation by drilling (see Fig. 9).

We then investigated another part of the study area where possible development of a deep talik was suggested by modelling results^16^. We conducted high-resolution seismic surveys along the same transect in two subsequent years. The top boundary of the observed acoustic anomaly moved upwards by ∼5 m in the course of just one year (2011–2012) (Fig. 8). It was thus concluded that the observed acoustic anomaly could not be ice within sediments; if it were, the position of the top ice boundary would have remained the same over the course of one year. In the marine environment, low-amplitude seismic anomalies, referred to as washed-out or semi-blanked zones, are associated with the presence of gas in sediments^28^,^46^; in permafrost, these anomalies may also result from physical property variations or changes associated with talik development^29^,^47^. It was shown that gas usually accumulates in the central portion of the channels presented by unconsolidated sediments held within consolidated sediments^28^. In permafrost areas, unfrozen sediments within taliks could represent such unconsolidated (unfrozen) sediments within consolidated (frozen) sediments. It is thus logical to expect that gas would accumulate and propagate in the central part of the talik, which is also what was observed at the reported site (Fig. 7). Finally, the velocity at which gas appears to be ascending is consistent with Darcy’s law (∼7 m year^−1^) assuming typical values for marine sediment permeability to gas, gas viscosity and gas density, suggesting that gas is propagating within the thawed/cryotic sediments. This also excludes the presence of ice-bonded permafrost in the sediments.

**Figure 9:**
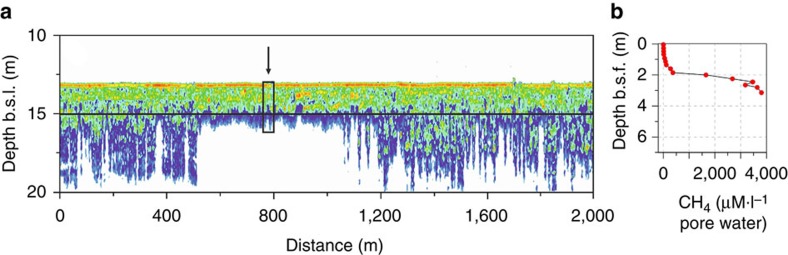
Results of validation of the acoustic anomaly observed in the study areas. (**a**) A high-resolution sub-bottom profile image revealing semi-blanked zone within the sediments; black arrow pointing to the black rectangle shows position of the borehole drilled to validate the observed acoustic anomaly; (**b**) change with depth in gas content observed in the recovered sediment core. The concentration of gas below the boundary identifying the acoustic anomaly increased by two orders of magnitude, allowing us to interpret the observed acoustic anomaly as being due to the presence of gas in the sediments.

To distinguish between subsea permafrost physical properties such as water/ice and gas within sediments, we validated one such acoustic anomaly by drilling a borehole at the site where such an acoustic anomaly was observed and measuring the gas content above the acoustic anomaly and within it (Fig. 9a). Within the observed acoustic anomaly, we measured an increase in CH_4_ concentration by two orders of magnitude (Fig. 9b). These data allowed us to interpret the observed acoustic anomaly as gas incorporated within sediments. Multiple pockmarks and bubbles propagating into the water column overlying the seafloor also point to gas releases occurring in the study area (Supplementary Fig. 8).

## Discussion

The stability of subsea permafrost underlying the ESAS near-shore zone is a key to whether pre-formed CH_4_ escapes from seabed reservoirs, or whether CH_4_ release is fuelled by modern methanogenesis occurring in recently-accumulated Holocene sediments and/or in thawed top permafrost layers^48^,^49^. Knowledge about the processes responsible for permafrost degradation after submergence by seawater was until recently mainly based on modelling results^5^,^7^,^9^,^18^. Three major assumptions were used to predict the current ESAS subsea permafrost state: (1) it degrades only upwards (bottom-up); (2) submerged near-shore zone thaw-lake taliks freeze after inundation; and (3) permafrost consists of non-saline sediments, thus the phase transition temperature is 0 °C. These factors combined would make permafrost to remain stable after submergence by seawater and to keep its integrity down to a few hundred metres, which would exclude any mass transfer including gas release^8^,^9^,^12^.

When the ESAS was exposed above sea level in cold-climate epochs, permafrost and permafrost-related hydrate deposits formed under very low temperatures^50^,^51^,^52^. After inundation, the bottom seawater temperature is only slightly <0 °C that is by up to 17 °C warmer than before inundation. Warming causes destabilization of subsea permafrost and permafrost-associated hydrates release some fraction of CH_4_ as free gas^5^,^9^,^19^. Because conversion of hydrates to free gas greatly increases the gas volume (by ≤200 times), accumulations of over-pressured gas tend to move upwards where sediment permeability allows this to occur, or where gas-migration pathways, such as taliks, are available^16^,^53^,^54^. Numerous gas blowouts followed by long-lasting gas flow have been reported from permafrost areas disturbed by exploratory drilling in Siberia, both on-land and offshore^3^,^22^,^52^. Such gas blowouts were reported from shallow permafrost-related gas-hydrate accumulations at depths of only a few tens of metres, starting from 20 m depth^55^.

Offshore, a particularly powerful gas discharge erupting from a well drilled through the subsea permafrost was documented in the Pechora Sea shelf; a gas–water fountain originating from 50 m beneath the sediment surface in 64 m-deep water reached 10 m above the ship. Echo sounding carried out at the drilling site 10 days after this event revealed an underwater fountain ∼10 m in diameter, with a height ∼40 m above the sea floor^56^. In the 1960s, gas was sampled from another blowout in the Yana River fore-delta at the Laptev Sea edge. Chemical analysis revealed high CH_4_ content (38.6%) in the sampled gas accompanied by significant helium and argon amounts, indicative of the deep origin of this gas^56^. Another document describes a blue burning gas flame observed on the water surface after sea ice in the DLS was demolished for navigation purposes^57^.

It was suggested by modelling that the time required for complete destabilization, according to modelling results, is ∼5–7 kyr, depending on time since inundation relative to previous freezing duration^4^. However, recent studies show that in some areas very recently (<<1 kyr ago) submerged permafrost is close to or has already reached the thaw point^17^,^58^. In a series of Pleistocene ocean transgressions and regressions, permafrost in the near-shore ESAS zone was suggested to have experienced an alternation of freezing and thawing, in consequence storing within it a large amount of hypersaline brines (called cryopegs)^21^. Salt from these accumulations is an effective corrosive factor that not only chemically disintegrates permafrost, but also alters its physical properties by decreasing its thaw point^13^,^51^. Downward propagation of salt ions from seawater and cryopegs could allow permafrost to remain ice-bearing or ice-free at temperatures <0 °C (ref. 15). Salt redistribution and downward movement could occur at speeds of at least 2 cm d^−1^ (refs 14, 59).

Salt rejected during freezing is transported into the thawed region by a convective mechanism, which is probably gravity-driven convection by salt fingering. It was shown experimentally that salt fingers could move with velocities of several centimetres per hour, and it was suggested that this could be a major mechanism for rapid salt movement in subsea permafrost^15^. Thaw of ice wedges within the submerged ICs could result in thermokarst depressions as deep as the thickness of those ice wedges, creating taliks. Further, downward development of taliks, along with upward degradation of underlying permafrost caused by geothermal flux, can lead to penetration of the entire permafrost body. Therefore, coastal ICs in the near-shore zone of the ESAS could be partially or completely destroyed by heat transfer and salt intrusion, and permafrost remains could represent subsequent layers of non-mineralized and mineralized sediments, thawed/cryotic and frozen^16^,^33^.

One possible mechanism allowing efficient gas-migration path formation in otherwise continuous permafrost is further downward development of pre-existing thaw-lake taliks, which were submerged during sea transgression^53^. Such thaw lakes were abundantly distributed on the Laptev Sea coastal plain; their possible interaction with the ocean has been described^4^,^9^. However, the authors assumed that after submergence by the sea, these taliks would freeze and stop developing downwards^8^,^9^,^12^. Our data show that this may not always be true. Submerged taliks may keep developing downwards; we likely observed this in the DLS (Laptev Sea). When DLS areas were exposed, ICs reached ≤50 m in thickness. When natural warming over this area started ∼15 kyr ago, thermokarst processes began to destroy ICs. Because ICs primarily consist of monolithic, wedge-like ice with ground material inclusions, when thermokarst starts it first destroys ice wedges in the IC structure. The permafrost thermal state is not the sole critical determinant of permafrost permeability for gas. Even ice-bonded permafrost is gas-permeable, especially when gas is over-pressured to enable it to build its own gas-migration pathways (like gas chimneys)^60^. One such possibility by which submerged thaw-lake taliks could serve as gas-migration paths is via a pingo-like structure, which might form through the taliks^61^.

Summarizing our results, we suggest that subsea permafrost degradation in the near-shore zone of the ESAS is determined by a combination of heat/salt-transfer and erosional processes, including thermokarst and collapse of submerged ICs, which is specific for the near-shore ESAS. Seafloor erosion could start from initial ground ice melt followed by formation of a polygonal form of seafloor landscape. It could further, or in parallel, be affected by chemical erosion caused by salt penetration into the frozen ground followed by removal of eroded material by bottom currents in the near-shore ESAS zone. Development of taliks of different origin could lead to formation of vertical erosion channels, helping gas fronts to propagate upwards towards the seafloor; otherwise, gas fronts tend to dome up towards the seafloor only at morphologic highs and/or at gas collector fronts and sides^62^. Also, ice scouring could unroof upper sediment layers, opening gas-migration pathways for underlying gas. The resulting gas release from underlying gas accumulations causes formation of ubiquitously observed pockmarks and other signs of sediment erosion and associated sediment settlement.

This area is strongly affected by Laptev Sea Rifts, which consist of several deep, seismically subsided and tectonically active rift basins^45^,^63^. The Lena River and coastal erosion are considered the main sediment sources to the Laptev Sea; sedimentation flow varies significantly, and sedimentation rates vary by orders of spatial and temporal magnitude through the year^34^,^35^,^36^. The pulsatile flow associated with spring ice breakup and after-breakup flooding causes progressive erosion of floodplain deposits and catchment areas^64^,^65^. It was shown that slight changes in seafloor erosion and sedimentation patterns that change the thermal and pressure regime below the seafloor could be viable mechanisms for unroofing underlying gas reservoirs, which can release CH_4_ in large quantities^66^. Once initiated, erosion could propagate further downward and migrate laterally to adjacent areas, driven by venting gas. Erosion of a few tens of seafloor metres could unroof over-pressured shallow gas reservoirs and buoyant hydrate-laden sediment accumulations beneath the seafloor, triggering rapid gas release^66^,^67^. Taken together, these processes could explain why the permafrost underneath the seawater is degrading more rapidly than its terrestrial sibling in the late Holocene. These observational constraints on both mechanisms, and rates of thawing and degradation of subsea permafrost on the ESAS provide a foundation for predictions of the future trajectory of CH_4_ release from this dynamic Arctic system.

## Methods

### Permafrost drilling and core processing

A heavy drilling technique was used to drill the subsea permafrost in the study area in annual spring campaigns (March–April 2011–2014). Drilling rig, well tubes, borehole casing and additional equipment were delivered to Tiksi (Yakutia, Russian Federation) by cargo air freighters (IL-76s). A caravan, which travelled over the fast ice to the drilling location, delivered equipment to the ice camp. The drilling was performed from 2 m-thick sea ice using a drilling rig (URB-4T) with a hydraulic rotary-pressure mechanism operating without drilling fluid (dry drilling) to avoid contamination. Well tubes and borehole casings 4 m long and 147 mm in diameter were used to preserve undisturbed core structure and prevent seawater infiltration. The casing was drilled through the sea ice, the water column and into the seabed sediments. After extraction from the borehole, sectioned sediment cores were cleaned, inventoried, packed in thermo-insulated boxes, delivered to the permafrost tunnel in Tiksi and stored at −12 °C. To deliver the sediment cores to shore-based laboratories, we chartered an AN-12 aircraft from Tiksi to Vladivostok; sediments were kept frozen (−20 °C) on board and on the refrigerator truck, which delivered frozen samples to the laboratory freezers, where they are kept at −18 °C. Surface sediment samples were retrieved with a Van Veen grab sampler. The topmost surface sediment layer was transferred with stainless steel spatulas to pre-cleaned polyethylene containers before being stored at −18 °C until analysis.

### Drilled borehole localization

During the 1982/83 drilling campaign, boreholes were localized using a shore-based theodolite technique. A tripod-mounted optical theodolite was precisely placed vertically using an optical plummet, and levelled over a ground mark using precise tubular spirit bubbles. Two long-established signs (the navigation sign for Cape Muostakh, Bykovsky Peninsula and the topography sign for Cape North, MI) were used as targets to measure horizontal angles during the theodolite survey. The theodolite technique allowed measurement accuracy to be better than ±0.5 m. Boreholes were drilled along the transect marked by 25 landmarks situated 25–50 m apart from each other. The position of each borehole and the entire transect direction were identified relative to the basic theodolite sign positions. Between the two drilling campaigns (1982 and 2013/2014), coastline dynamics were observed using theodolites, remote-sensing techniques and *in situ* observations. The mean annual northern MI coastal retreat rate was 6 m year^−1^ (refs 11, 35). In 2011/2014, borehole positions for re-drilling were identified using GPS. Boreholes 301 (4D-14), 303 (4D-13), 304 (3D-14) and 305 (2D-13) were drilled 300, 600, 850 and 2,500 m, respectively, from the 1982 coastline position.

### Time since inundation

To reconstruct the inundation history of the Laptev Sea shelf, some authors performed a reconstruction based on major changes in average sedimentation rates using radiocarbon dating of the available sediment cores^36^. Other authors proposed a conceptual model of permafrost transition in the coastal zone based on glacial history and sea level variation in eastern Siberia^4^. Specifically, during the last 5 thousand years (5 kyr), after the highstand of the Holocene transgression was reached, submergence of the eastern Siberia near-shore zone has been occurring due to erosion-fed processes^36^. Before 5 kyr, the shoreline shifted by 300–800 km southward^36^. Thereafter, the transformation of the coastline, in particular in Buor-Khaya Bay, was due to active thermal erosion of the submerged IC. An IC is an ice-rich deposit with massive ice wedges, which might include >80% gravimetric ice content^35^. IC erosion led to the disappearance of the bridge connecting the Bykovsky Peninsula and its southernmost part, which became MI. In the Laptev Sea region, 25% of the 7,500-km-long coastline is composed of ICs. Heat transfer to the ground ice exposed along the permafrost-dominated coasts combined with the mechanical force of waves and wind led to rapid thermo-denudation and thermo-abrasion, resulting in coastal retreat accompanied by coastal landslides, mud stream development and delivery of the clastic material to the sea bottom^23^. The modern shoreline retreat rate was suggested to vary between 2 and 25 m year^−1^ on the northern part of MI^23^,^35^.

To assess the time since inundation of the drilled boreholes, we applied an empirical modelling approach based on observational time series. Our major assumption followed^36^, who suggested that by 5 kyr, sea level in this region achieved its highstand between the 30 and 20 m isobaths. The fraction of the shelf shallower than 20 m, ∼30 % of the total area of the ESAS, was taken by the ocean owing to the erosion-based processes of thermo-denudation and thermo-abrasion. Therefore, between two subsequent isobaths the water depth adjusted to the currently existing bathymetry at a speed that depended on the rates of coastal erosion and the vertical angular difference between horizontal planes located at two subsequent isobaths (inclination, Supplementary Fig. 5). We used a bathymetry map of the area updated with the results of our multi-year *in situ* investigations to apply a digital elevation model using Surfer-11. The near-shore area surrounding MI was gridded using a relatively even grid cell net to achieve detailed coverage of the northern part of MI where the drilling was performed. The rate of coastal erosion set for this approach was based on observational data collected during the regular stations’ monitoring of coastal erosion on MI conducted during the last 62 years. These time series documented seasonal variability in rates of coastal erosion on MI of up to 25 myear^−1^, which was observed in 2005; rates varied within a wide range of values. However, it was documented that from 1983 to 2013, the position of the coastline shifted inland by ≈180 m, establishing a mean annual coastal erosion rate of 6 m year^−1^ following^11^. This rate was applied to estimate time since inundation using the following algorithm.

To calculate time since inundation at four re-drilled boreholes, we assumed that exclusively erosion-fed processes such as thermo-abrasion and thermo-denudation determined inundation in this near-shore area. We also assumed that no actual sea level rise and sediment accumulation occurred along this particular transect. This assumption is based on the findings that Holocene-age sediments are absent in the top layers of the drilled sediment cores, that coarse-sized grains are prevalent in the sediment granulometric structure, and that sands and silty sands predominate in the lithological structure of the top sediments (according to the Shepard ternary diagram^68^, Supplementary Fig. 1).

Time since inundation *T* depends on speed of coastal erosion, distances between isobaths, and the steepness of the sea-floor slope (Supplementary Fig. 5). Sea level adjustment between two subsequent isobaths *R* is described by *R*=*C* sin(*A*), where *C* is distance between two subsequent isobaths, and *A* is inclination, or the slope angle between two subsequent isobaths. Because we drilled in the very shallow near-shore zone (water depth <5 m), we calculated inclination for isobaths at the 1, 2, 3, 4 and 5 m water depths; in this case, sin(*A*)=1/*C.* Each year the sea level was adjusted by a fraction *R*_*i*_ which is determined by mean annual rate of coastal erosion *C*_*i*_ and inclination *A* so that: *R*_*i*_=*C*_*i*_ sin(*A*); correspondingly, *R*_*i+*1_*=C*_*i+*1_sin(*A*); *R*_*i+*2_=*C*_*i+*2_sin(*A*)*; R*_*i+n*_=*C*_*i+n*_sin(*A*). We assumed mean annual rate of coastal erosion to be equal during all times of inundation, *R*_*i*_=*R*_*i+*1_*=R*_*i+n*_; therefore, the number of years *T* required for the ocean to adjust between two subsequent isobaths is *T*=*R*/*R*_*i*_. Knowing the distance between the location of the coastline in 1982–1983 and each of the four drilled boreholes (300, 600, 850 and 2,500 m), we located the position of the boreholes between different isobaths and calculated the time from inundation to 1982–1983 for each borehole starting from the nearest borehole to the shore (Supplementary Fig. 6). The time since inundation of the next borehole positioned between two isobaths was calculated as follows. Time since inundation for the previous borehole was summarized with a fraction of the time required for the sea level to adjust to the water depth of the next isobath, which is proportional to the distance from previous isobaths to the borehole. The coastal erosion rate was set at 6 m year^−1^. The calculated times since inundation for the four boreholes are 48, 128, 200 and 442 years, correspondingly, for boreholes 4D-14, 4D-13, 3D-14 and 2D-13. Time since inundation for borehole 4D-12 was calculated to be 477 years.

We also applied another approach, which is based on the distance from the current shoreline to each borehole along the transect (300 m for borehole 4D-14, 600 m for borehole 4D-13, 850 m for borehole 3D-14, 2,500 m for borehole 2D-13 and 2,900 m for borehole 4D-12). We converted the distance from the shore to the borehole by assuming that the retreat rate of 6 m year^−1^ remained the same since the time of inundation. This method equals directly measuring the adjacent leg of the triangle in Supplementary Fig. 5 instead of calculating it as described above. The calculated times since inundation for the four boreholes are 50, 100, 141 and 416 years, correspondingly, for boreholes 4D-14, 4D-13, 3D-14 and 2D-13. Time since inundation for borehole 4D-12 was calculated to be 484 years. Calculation of the uncertainties of our estimates performed by these two methods allowed us to put an error range to the pre-1982 rates of permafrost degradation (see columns g and h in Table 1).

### Thermal borehole measurements

Each borehole’s temperature was recorded for ∼3 days after drilling using a chain of calibrated hydro-isolated thermistors, following Global Terrestrial Network for Permafrost (GTN-P, http://gtnp.arcticportal.org/component/content/article/19-data/mining/80-protocols-good-work-practices) protocol. Chain length depended on borehole depth and varied from 20–50 m; thermistor numbers depended on borehole depth, with a thermistor installed for each 2–4 m of the borehole. Recordings were performed every 30 min; measurement accuracy was ±0.01 °C. A temperature logger (HOBO Temp PRO V2) was installed in the probe head. To prevent the chain from adhering to the borehole walls, we covered it with plastic tubing. Data were tested statistically using an empirical distribution function test in the Statistics 7.0 software package. Descriptive statistics were calculated for the 95% confidence interval of the mean (*P*=0.95, alpha=0.05). Only data points complying with a statistically-established threshold (s.d.) of 0.05 °C were reported.

### High-resolution sub-bottom profiling

Hydro-acoustical and high-resolution seismic data were recorded in 2008, 2011 and 2012 for a distance of ∼3,370 nautical miles. In 2008 and 2011, high-resolution seismic sub-bottom profiles were collected using a chirp sonar system, which consisted of a GeoPulse Subbottom Profiler (Model 5430A) equipped with a Model 136A tow fish. This system operates in a tuned-frequency range of 2–12 kHz; it carries four mounted wide-band piston-type transducers (Model T135) and a ceramic line array as the acoustic receiver (GeoPulse Receiver, Model 5210A). The chirp sonar system emits a computer-generated frequency-modulated swept pulse with the 2–12 kHz frequency band as source signal. To perform real-time correlation processing, the acoustic signal was digitally recorded (SEGY format) in a deck unit where the pulse transmitter and a correlation filter were installed. Returning signals were recorded by a RoverBook G4 PC using GeoPro-2 software, and were processed using a matched-filter correlation technique to collect the 2–12 kHz frequency band, which provides higher-resolution images than does the 3.5 kHz system. Navigation was controlled by a differential GPS (GARMIN Model 120 XL) with ≈10 m accuracy. The vessel speed during recording on polygons was 4–6 nautical knots, and between polygons 5–12 nautical knots. A chirp pulse varied from 50–500 ms. A 100 ms-long chirp pulse was mainly used with 3.5 kHz frequency (resolution of ∼0.5 ms) resulting in 40 ms and 90 ms penetration (acoustic two-way travel times, TWTs), corresponding to 30–70 m sediment thickness. High-resolution seismic data were analysed using the GeoPro-2 software package and interpreted following^28^.

In 2011 and 2012, in addition to a GeoPulse Subbottom Profiler, a new parametric echo sounder (SES-2,000 Standard) with penetration depth of ≤50 m (accuracy ±0.02 m) and primary transmitter frequency of 100 kHz was used to create an accurate picture of the bottom layer and the sediment structures beneath it. Because of high-parametric system bandwidth, short signals can be transmitted. This makes parametric systems particularly useful in shallow-water areas, enabling detection of small changes in acoustical impedance. The signal processing was digital and produced real-time colour echo plots, allowing quick survey adaptations. Digitally storing echo-sounder data together with attached positioning-system data allows complete post-processing using the ISE-2.9.2 software package (http://innomar.com) for further visualization, charting and volume calculation.

### Backscatter data

The bathymetric survey was performed using the WASSP WMB-3250, a multi-beam swath sonar system operated with a frequency of 160 kHz, a resolution of 224 beams per ping and an opening angle of 120°, which results in a swath width of 3–4 times the water depth. Sound speed profiles of the water column were acquired using conductivity-temperature density (CTD) data and sound velocity profiler (SVP) data collected using the SWiFT SVP (Valeport, UK) equipped with integral GPS to geo-locate every profile. Data were downloaded, reviewed and translated to common SVP formats wirelessly via Bluetooth Smart using the SWiFT APP. Standard DataLog X2 software for PC was used to support SWiFT. Bathymetric data were acquired and processed using hydrographic survey software Hypack (HYPACK). Grids with optimized resolutions, depending on the water depths of mapped areas, were created for the analysis of bed forms. Seafloor morphology was interpreted in the 3D environment of WASSP Navigator and Fledermaus software. Side-scan sonar data were collected using the 2638A Hydra Series III Data Acquisition System (FLUKE Calibration, US), equipped with 250 and 500 kHz transmitters and a flexible 22-channel Universal Input Connector, which allowed connection with digital data storage with multi-channel real-time data display (DC accuracy of 0.0024%; thermocouple accuracy of 0.5 °C; full-colour display). Side-scan sonar data were acquired using the Hypack software.

### Bubble detection in the water column

To detect water column bubbles, sonar data were gathered using a SIMRAD EK 15 SW 1.0.0 echo sounder (www.simrad.com) with 200 kHz operational frequency, 80–1,240 μs pulse duration, 26° beam width, and a built-in calibration system. Data were recorded at an average 4–6 knots survey speed and visualized and processed using an original software package provided by SIMRAD EK 15 (EchoView and Sonar5). Details are available elsewhere^16^.

### Statistical dataset testing

Data were tested statistically using an empirical distribution function test in the Statistics 7.0 software package. Descriptive statistics were calculated for the 95% confidence interval of the mean (*P*=0.95, alpha=0.05). Discussion of uncertainties associated with applied methods can be found in the Supplementary Information.

### Cryo-stratigraphy and granulometry

The sediment cryostructure was described following a published protocol^69^. Size composition of fine-grained sediments and suspended particulate matter was studied using a laser microanalyser (Analysette 22, Fritsch GmbH). Sieve analysis was used for examining coarse sediments^34^. Bottom sediments were then sized on the basis of a three-component classification^68^ with definitions: sand (>0.1 mm), silt (0.1–0.01 mm) and clay (<0.01 mm).

### Geoelectrical data

Several geoelectrical surveys were conducted from the fast ice in the Buor-Khaya Bay, Laptev Sea (2012–2015). We used the pulled array transient electromagnetic method (PATEM). The PATEM system provides high-lateral resolution and high-data density to ensure data quality. When measurements are performed continuously in time and space, variability in data not caused by changes in geology are clearly visible. A sounding consisted of an array, which composed of a 25 × 25 m transmitting heavy-duty loop, which used a transmitted current of 20 A with a total period of 10^−5^ s, and centrally positioned enclosed a 20 × 20 m receiver loop, which measured the incoming signal. The transmitter and receiver were framed and towed along the studied transects by an all-terrain vehicle or a caterpillar. The array position was permanently located using a GPS; the injection current and electrode pair potentials were recorded continuously as the array was towed. Data were inspected and filtered using a running mean. To interpret the sounding curve, we used software Faraday, which is modified version of the Dipole1D and Occam1D inversion^32^,^70^. The modelled domain represented a few-layer horizontally bedded medium. Inversion performance was measured by calculating the root mean square error between the measured and modelled apparent resistivity.

### Data availability

The data reported in this paper are archived in the Bolin Centre for Climate Research Data Archive (http://bolin.su.se/data/).

## Additional information

**How to cite this article:** Shakhova, N. *et al*. Current rates and mechanisms of subsea permafrost degradation in the East Siberian Arctic Shelf. *Nat. Commun.* **8,** 15872 doi: 10.1038/ncomms15872 (2017).

**Publisher’s note:** Springer Nature remains neutral with regard to jurisdictional claims in published maps and institutional affiliations.

## Supplementary Material

Supplementary Information

## Figures and Tables

**Table 1:** Rates of ice-bonded permafrost table deepening in the study area.

**Boreholes 1982/83 versus 2013/14**	**Latitude °N**	**Longitude °E**	**Water depth (m.b.s.l.)**	**Core length (m)**	Depth of IBPT* **1982 (m.b.s.l.)**	**Years since inundation to 1982**†	**Rate of IBPTD cm per year before 1982**	**Depth of IBPT 2013/14 (m.b.s.l.)**	**Change in depth of IBPT from 1982 to 2013/14**‡	**Rate of IBPTD cm per year from 1982/83 to 2013/14**§
**a**	**b**	**c**	**d**	**e**	**f**	**g**	**h**	**i**	**j**	**k**
301/4D-14	71° 36′	129° 55′	2.5	9.5	4.2 (3.3)	49±0.7	8.5±0.02	8.6	4.4 (5.3)	15.1±1.6
303/4D-13	71° 37′	129° 55′	2.5	21	5.8 (7)	114±10	5.2±0.38	11.4	5.6 (4.4)	18.3±0.1
304/3D-14	71° 37′	129° 54′	2.7	17.6	8.6 (8.3)	170±21.7	5.2±0.6	12.8	4.2 (4.5)	13.5±0.2
305/2D-13	71° 37′	129° 52′	3.4	30.4	16.8 (16)	429±9.2	3.9±0.01	19.3	2.5 (3.3)	9.3±1.7

IBPT, Ice-bonded permafrost table.

S.e.m. in columns h and k reflects variability between values calculated from the two groups of numbers presented in columns f and j.

^*^Data presented in column f are from^20^; numbers in parentheses are original data from drilling report provided by Grigoriev.

^†^Years since inundation calculated as described in Methods section.

^‡^Numbers in parentheses calculated from values presented in parentheses in column f.

^§^Rates of IBPTD from 1982 to 2013/2014 derived from j*/*31 for boreholes 4D-13 and 2D-13 or j*/*32 for boreholes 4D-14 and 3D-14.
