# Supplementary material for: Current rates and mechanisms of subsea permafrost degradation in the East Siberian Arctic Shelf
Source: Nat Commun. 2017 Jun 22;8:15872. doi: 10.1038/ncomms15872 (PMC5489687; doi:10.1038/ncomms15872)
Supplement: Supplementary Information [file ncomms15872-s1.pdf]

SI Guide

Type of file: pdf
Size of file: 0 KB
Title of file for HTML: Supplementary Information
Description: Supplementary Figures, Supplementary Tables, Supplementary Discussion, and Supplementary References

SUPPLEMENTARY FIGURES

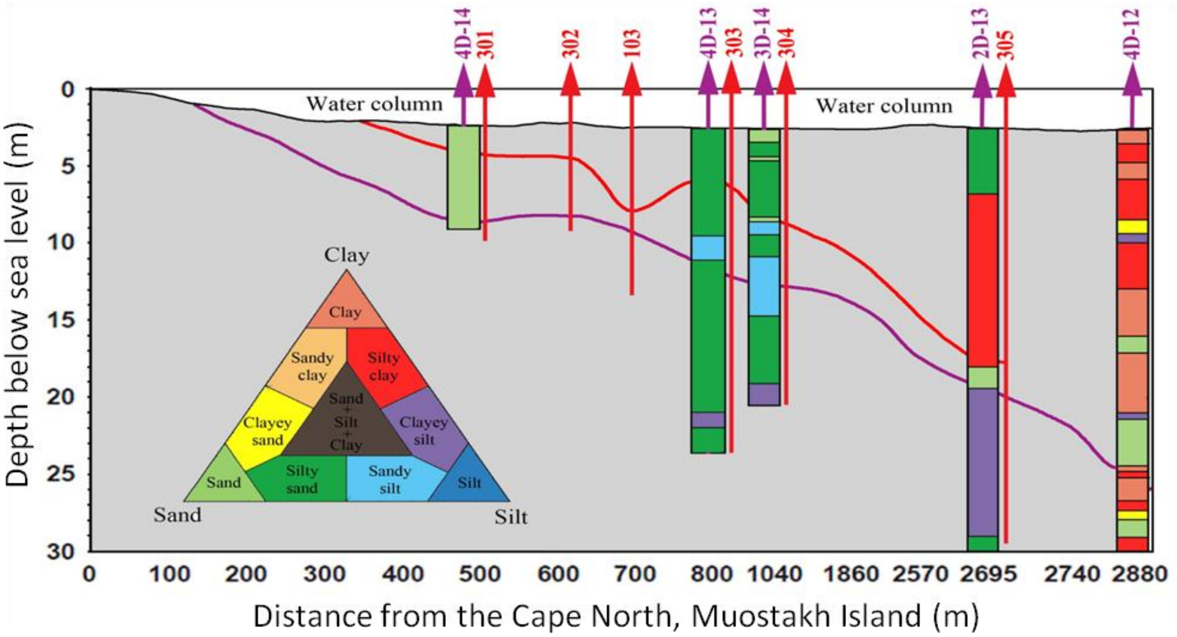

Supplementary Figure 1 | Lithology of the sediment cores recovered in the near-shore zone of the East Siberian Arctic Shelf (ESAS). Based on the proportions of sand-, silt- and clay-sized particles, the bottom sediments were classified according to Shepard's diagram[1]. Shepard's diagram is an example of a ternary diagram divided into ten classes and follows the conventions of all ternary diagrams; it is a three-component system summing to 100%. For example, Shepard's "Clays" contain at least 75% clay-sized particles, "Silty Sands" and "Sandy Silts" contain no more than 20% clay-sized particles, and "Sand-Silt-Clays" contain at least 20% of each of the three components. Purple line, connecting the sea floor and the IBPT documented in the drilled boreholes, marks suggested position of the IBPT in the sediments based on interpretation of validated geo-electrical data (presented in Supplementary Fig. 4).

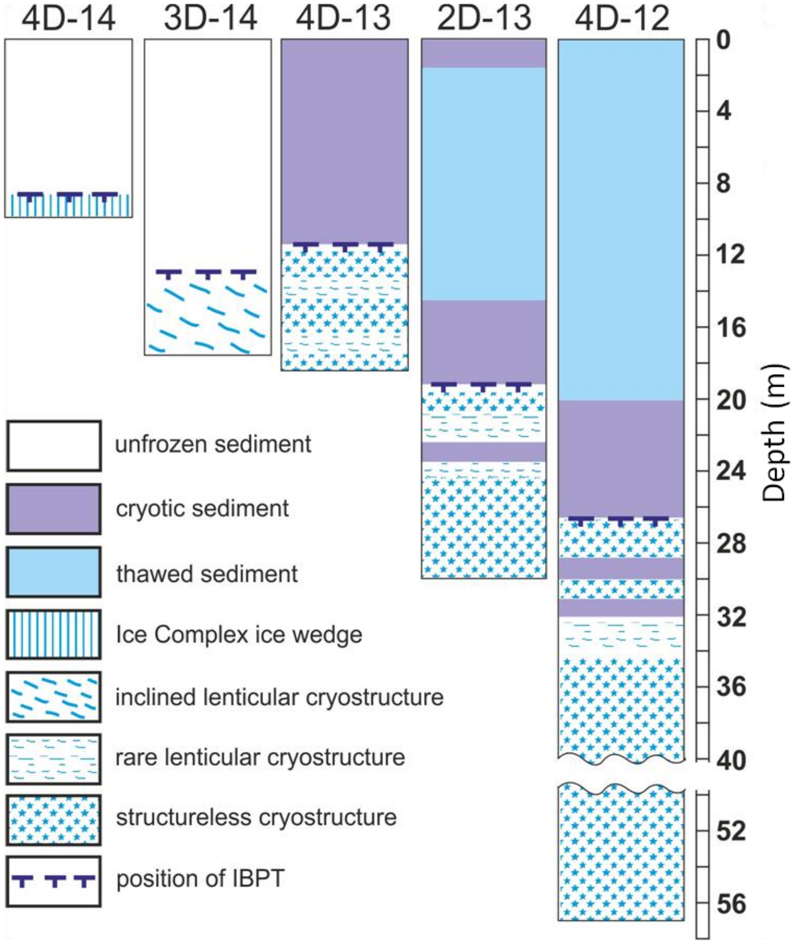

Supplementary Figure 2 | Cryo-stratigraphy of the sediment cores recovered in the study area. Thawed sediments are sediments at temperatures >0°C observed above the IBPT; cryotic sediments are sediments at temperatures <0°C observed above the IBPT; unfrozen sediments are sediments observed above the IBPT, in which temperatures were not measured for technical reasons. The lenticular cryostructure was characterized by clear presence of ice lenses within frozen sediments. The ice lenses themselves were usually a few millimeters thick and separated by closely-spaced, debris-rich to ice-rich, cryostructures or sediment bands. The volumetric ice content of some drilled boreholes varied from 17 to 62% (Supplementary Table 2).

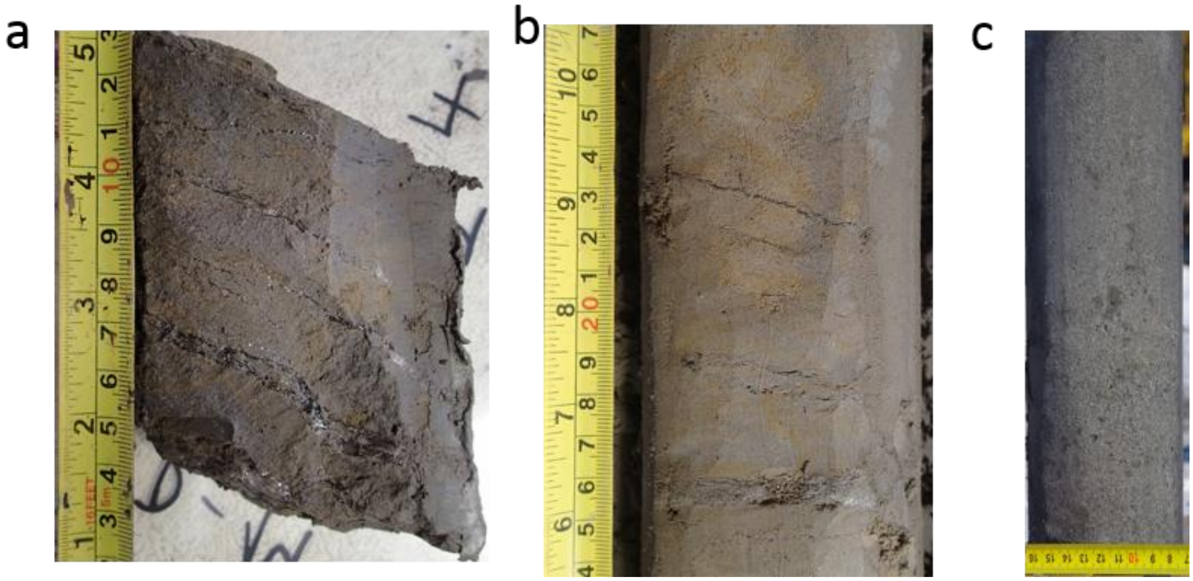

Supplementary Figure 3 | Examples of cryostructures observed in the recovered sediment cores. (a) An inclined lenticular cryostructure was characterized by clear presence of ice lenses within frozen sediments. The ice lenses themselves were usually a few millimeters thick and separated by closely-spaced, debris-rich to ice-rich, cryostructures or sediment bands; (**b)** A rare lenticular cryostructure was characterized by rare presence of ice lenses within frozen sediments; (**c)** The structureless cryostructure was characterized by millimeters size ice aggregates randomly distributed in the sediments.

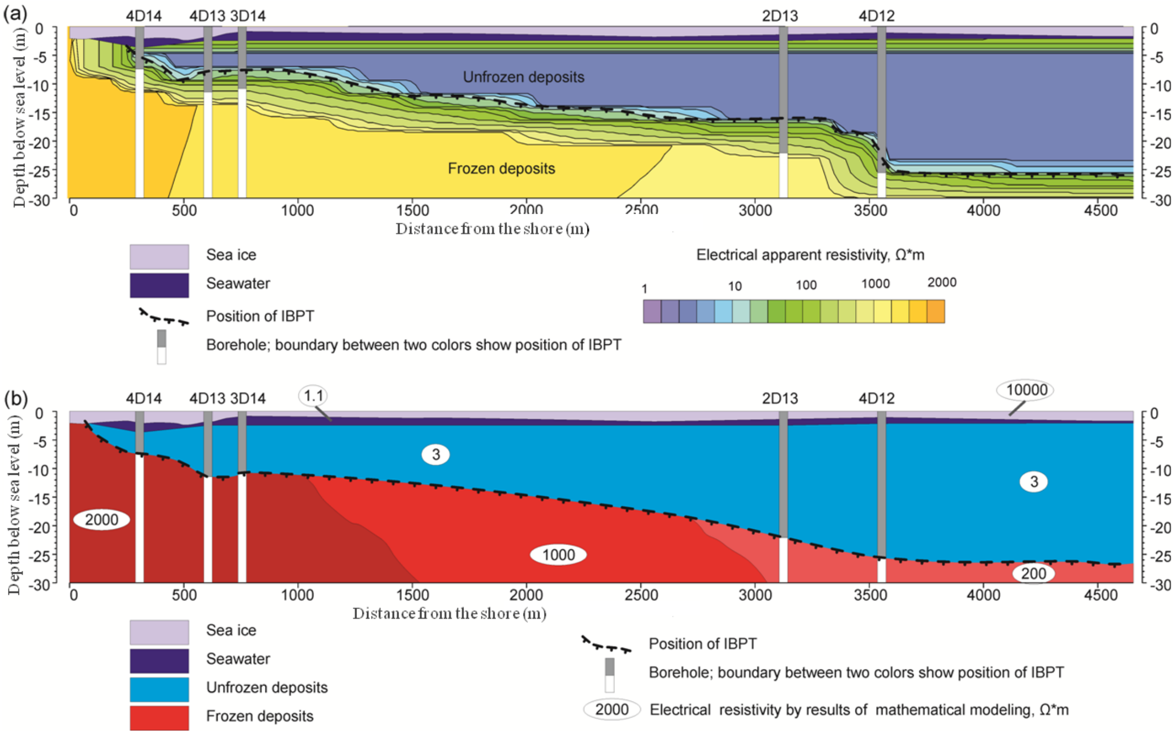

Supplementary Figure 4 | Results of inverse modeling of sub-bottom electrical resistivity along the drilling transect. (a) Computed depth of the ice-bonded permafrost table (IBPT) below sea level (b.s.l.) based on apparent electrical resistivity of sediments is shown as a black dotted line drawn between drilled boreholes. As seen from the panel, computed position of the IBPT does not agree with actual position of the IBPT observed in drilled boreholes; **(b)** computed depth of the IBPT based on measured actual electrical resistivity of recovered sediments is shown as a black dotted line drawn between drilled boreholes; as seen from the panel, computed position of the IBPT agrees well with actual position of the IBPT observed in drilled boreholes. Apparent resistivity data show variability in electrical resistivity from 2.3 to 1850 Ohm m^{-1}, while variability in real resistivity measured in the recovered sediment cores was from 2.1 to 1964 Ohm m^{-1} (Supplementary Table 1). Within thawed sediments real resistivity varied from 1 to 100 Ohm m^{-1} depending on salt content of the sediments; in frozen sediments, resistivity varied from 100 to 2000 Ohm m^{-1} depending on salt content of the sediments. Computation performed using the software Faraday. The theoretical and experimental parts of the algorithm is presented in Rivera et al.[2] and Dudarev et al.[3].

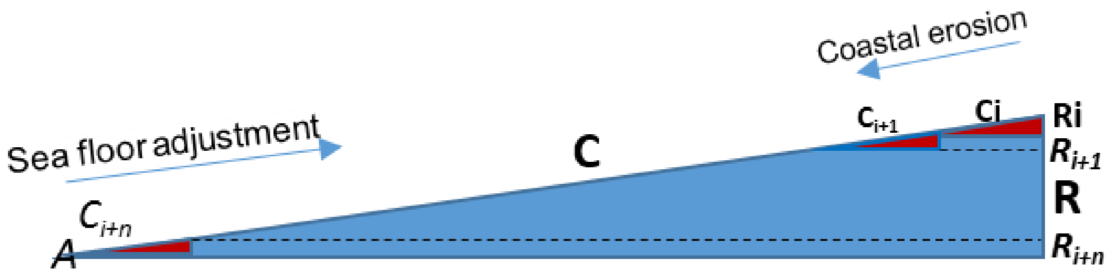

Supplementary Figure 5 | Schematic presentation of the algorithm used to calculate time since inundation in the re-drilled near-shore boreholes. ***C*** is the distance between two subsequent isobaths (1m, 2m, 3m, 4m, and 5m); $\boldsymbol{C_i}$ to $\boldsymbol{C_{i+n}}$ is fractions of the coastline taken by erosion in one year; ***A*** is inclination, that is the slope angle between two subsequent isobaths; ***R*** is sea level difference between two subsequent isobaths, which is 1 m in this case; $\boldsymbol{R_i}$ to $\boldsymbol{R_{i+n}}$ is fraction of the sea level by which it rises each year due to coastal erosion.

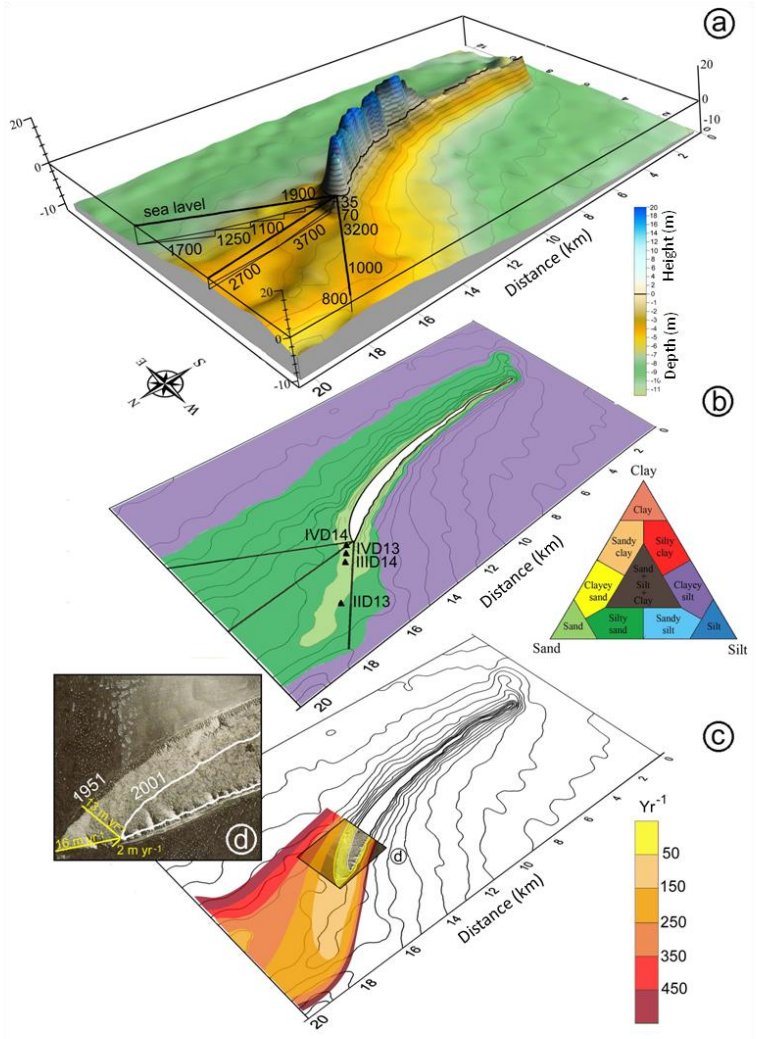

Supplementary Figure 6 | Results of a digital elevation model and specific features of sedimentology and coastal erosion in the study area. (**a)** Results of 3D modelling of the local bathymetry along the re-drilled transect; **(b)** sedimentology of the uppermost layer based on the Shepard ternary diagram; (**c)** time since inundation of the re-drilled transect and its vicinity based on results of empirical modelling; (**d)** aerial photographs showing positions of the coastline in 1951 and 2001 and consequent rates of coastal erosion in three different directions varying from 2 to 16 m yr^{-1}.

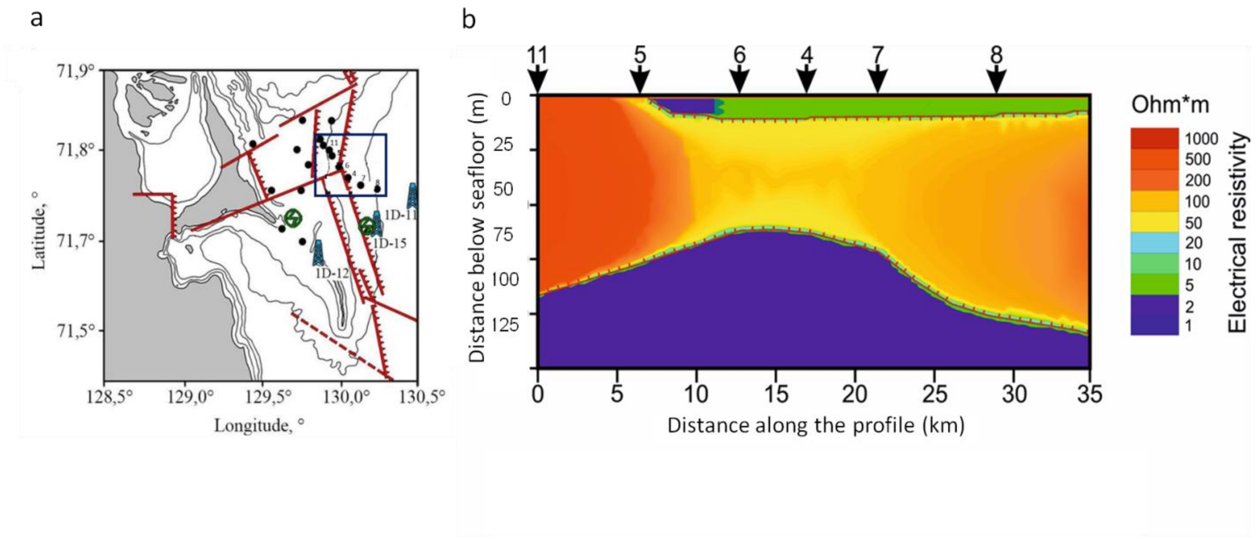

Supplementary Figure 7 | Inverse model of sub-bottom electrical resistivity for one transect performed in the study area. (a) Study area, in which geo-electric investigations were performed. Position of the transect presented in the panel **b** marked as a chain of black dots within the black rectangular marked in the panel **a**. Red saw-like lines refer to active faults with known kinematics; solid lines refer to position of active faults with unknown kinematics; red dotted line shows position of presumed faults; (**b)** Large variations in resistivity with distance and with depth and the configuration of the contours (yellow) correspond to the shape of the presumed boundaries of thawed/cryotic sediments observed along the transect Apparent resistivity between lines 11 and 7 (marked with black arrows) falls from 1000 Ohm m to 50 Ohm m and rises again after line 8, which points to significant variability in sediment properties along the transect and possible existence of a tectono-genetic talik.

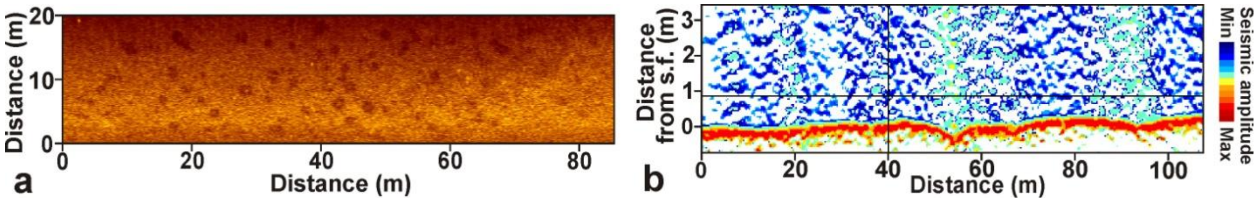

Supplementary Figure 8 | Specific morphological features of the seabed and bubble plumes observed in the water column. (a) A side-looking sonar image of a typical pockmark field observed in the sea floor in the study area; (**b)** a high-resolution sub-bottom profile image showing bubble plumes propagating from the sea floor in the area of investigation (shown in green); difference in colors between the water and bubbles reflects degree of sound wave refraction depending on amplitude variation associated with presence of gas bubbles in the water.

Supplementary Table 1 | Volumetric ice content of different cryostructures identified in the drilled boreholes.

**Borehole 4D-12**		**Borehole 2D-13**		**Borehole 5D-13**	
Depth (m)	**Volumetric ice content (%)**	**Depth (m)**	**Volumetric ice content (%)**	**Depth (m)**	**Volumetric ice content (%)**
29.9	40	17	29	8.3	50
31.9	46	18	29	11	33
32.9	35	19.3	30	11.8	62
34.1	27	19.6	29	13	33
35.6	27	21	27	23.5	33
37.4	36	22.3	34	24	34
39.0	38	23	24	27.8	35
42.4	40	24.2	27	28.4	31
45.2	39	25	18	30	34
47.2	36	25.5	17	30.5	33
50.0	40	26.3	30	33	34
50.8	40	26.9	29	34.1	30
54.1	42	27.3	55	38	32
		29	54	38.8	34
		29.7	31		
		30	24		
		31	23		
		31.5	31		
		32.2	33		

Supplementary Table 2 | Observational data used to develop the relationship between resistivity values and depth of sediments**.**

Borehole	Depth of sampling, m	Apparent resistivity (Ohm m)	Resistivity (Ohm m)	Position of IBPT** b.s.l., m; salinity*
2D-13	5.8	3.2	2.3	19.3
2D-13	10.4	3.4	2.8	
2D-13	24.6	830	550	Saline
3D-14	4.5	12	2.4	12.8
3D-14	14.5	1678	2045	Non-saline
4D-13	5.2	2.3	2.1	11.4
4D-13	9.5	87	2.5	
4D-13	15.4	1850	1430	Non-saline
4D-14	3.7	51	2.6	8.6
4D-14	8.2	1020	1964	Non-saline

*A comparison between inverted resistivity and actual resistivity was made between saline and non-saline sediments;
** IBPT stands for ice-bonded permafrost table.

SUPPLEMENTARY DISCUSSION

It was reported that rates of coastal erosion on the northern part of MI were higher than elsewhere on the island and ranged from 2 to 25 m in the summer season. If rates of coastal erosion were less than 6 m yr^{-1}, T_i would be longer (Supplementary Fig. 5 and 6); this would affect our estimates by further decreasing calculated rates of permafrost degradation during the time prior to drilling in 1982/83. If rates of coastal erosion were higher in the past, T_i would be shorter; this would increase calculated rates of permafrost inundation. However, rates of coastal erosion observed on the northern part of MI are the highest among coastal sites in the near-shore zone of the Laptev Sea; moreover, these rates were reported to be 1.9 times higher from 2013-2015 than they were from 1951-2012 (ref. 4). Thus, it is very unlikely that rates of coastal erosion in the studied area were ever higher than 6 m yr^{-1}. Therefore, we calculated conservatively that time since inundation for the drilled boreholes 301, 303, 304, and 305 were 48, 128, 200, and 442 years, respectively.

The Lena River is one of the two main sources of sediments for the Laptev Sea: the other is coastal erosion[5-8]. Previous studies reported a high proportion of fine-grained sediments in the Laptev Sea, with sandy deposits (<5% clay) restricted to northern areas of the Lena Delta[9,10]. The average sedimentation rates (ASR) in the Laptev Sea ranged from 0 cm kyr^{-1} in the areas with progressing fluvial erosion to 170 cm kyr^{-1} in the depocenters, which are 200-250 km north of the Lena Delta[8,9,11,12]. Near the Lena Delta, ASR were reported from 9 to 63.7 cm kyr^{-1}; the general understanding of sedimentary processes was that higher ASR occur closer to the Lena Delta[2]. Our study area is close to the Lena Delta and located southeast of the Bykovskaya branch of the Lena Delta, which carries ~25% of the Lena runoff. However, the Bykovsky Peninsula that lies between the Bykovskaya branch and MI partitions off the Bykovskaya branch outflow.

This natural barrier serves as a natural catchment for fluvial sediment flow north of the Bykovsky Peninsula, leaving the other part of the peninsula much less affected by river runoff. As was discussed above, results of lithological descriptions and granulometric analyses of the top layers of the sediment cores extracted from the re-drilled boreholes, unlike results reported in[2], revealed a predominance of coarse- and medium-grained sands of Pleistocene age with minor inclusions of silty sand resulting from redistribution by sea ice[3] (Supplementary Fig. 1). This points to the erosional rather than accumulative type of sedimentation along the studied transect.

SUPPLEMENTARY REFERENCES

1. Shepard, F.P. Nomenclature based on sand-silt-clay ratios. *J. Sediment. Petrol.* **24**, 151-158 (1954).
2. Rivera, J., Karabanov, E. B., Williams, D. F., Buchinskyi, V., & Kuzmin, M. Lena River discharge events in sediments of Laptev Sea, Russian Arctic. *Estuar. Coast. Shelf S.* **66,** 186-196 (2005).
3. Dudarev, O.V., Semiletov, I.P., Charkin, A.N. & Botsul, A.I. Deposition settings on the continental shelf of the East Siberian Sea. *Dokl. Earth Sci*. ***409***, 822-827 (2006).
4. Gunther, F., Overduin, P. P., Yakushina, I. A., Opel, T., Baranskaya, A. V., & Grigoriev, M.N.. Observing Moustakh disappear: Permafrost thaw subsidence and erosion of a ground-ice-rich island in response to arctic summer warming and sea ice reduction. *The Cryosphere* **9**, 151-178 (2015).
5. Alabyan, A. M., Chalov, R. S., Korotaev, V. N., Sidorchik, A. U., Zyatev, A. A. Natural and technological water and sediment supply to the Laptev Sea. *Berichte zur Polar- und Meeresforschung* **176**, 265-271 (1995).
6. Ivanov, V. V. and A. A. Piskun. Distribution of river water and suspended sediments in the river deltas of the Laptev Sea. *in Laptev Sea System* 142-153 (Berichte zur Polarforschung, 1995).
7. Kuptsov, V. M., & Lisitsin, A.P. Radiocarbon dating of Quaternary along shore and bottom deposits of the Lena and the Laptev Sea sediments. *Mar. Chem.* **53,** 301-311 (1996).

8. Are, F. E. The contribution of shore thermo-abrasion to the Laptev Sea sediment balance. *in Proceedings of the Seventh International Conference on permafrost* 25-30 (Yellowknife, Canada, 1998).

9. Bauch, H. A. *et al.* Chronology of the Holocene transgression at the North Siberian margin. *Glob. Planet. Change* **31,** 125-139 (2001).

10. Gukov, A. Y. *Hydrobiology of the Lena River mouth area (*Scientific World Press, Moscow, 2001).

11. Bauch, H. A., Kassens, H., Erlenkeuser, H., Grootes, P. M., Thriede, J. Depositional environment of the Laptev Sea (Arctic Siberia) during the Holocene. *Boreas* **28**, 194-204 (1999).

12. Vetrov, A. A., & Romankevich, E.A. *Carbon Cycle in the Russian Arctic Seas* (Springler-Verlag, Berlin Heidelberg, 2004).
